# LED Light Treatments Induce Activation of the Antioxidant Defense System in *Thymus mastichina* L.

**DOI:** 10.3390/plants14182930

**Published:** 2025-09-20

**Authors:** Gustavo J. Cáceres-Cevallos, Almudena Bayo-Canha, María Quílez, María J. Jordán

**Affiliations:** Research Group on Rainfed Agriculture for Rural Development, Department of Rural Development, Oenology and Sustainable Agriculture, Murcia Institute of Agri-Food and Environmental Research (IMIDA), La Alberca de las Torres, 30150 Murcia, Spain; gustavoj.caceres@carm.es (G.J.C.-C.); almudena.bayo@carm.es (A.B.-C.); maria.quilez@carm.es (M.Q.)

**Keywords:** *Thymus mastichina*, Spanish marjoram, LED light spectra, photosynthetic pigments, SOD, CAT, tocopherols, polyphenols, oxidative damage

## Abstract

This study investigated how different spectral ranges of LED light affect the synthesis of photosynthetic pigments and antioxidant systems in *Thymus mastichina* L., focusing on two ecotypes with distinct chemotypes: linalool and eucalyptol. The ecotypes were exposed to white, red, blue, red-blue (70:30), white-blue, or white-red light for 30 days under a 16/8 h light/dark cycle (115 μmol/m^2^s). Photosynthetic pigment content, lipid oxidative damage, antioxidant capacities, and both enzymatic (SOD, CAT) and non-enzymatic (tocopherols and polyphenols) antioxidant systems were assessed. For the linalool chemotype, red-blue light significantly increased carotenoid content, antioxidant capacity, and catalase activity, while elevating levels of plastochromanol-8 and phenolic compounds such as salvianolic acid B, rosmarinic acid, and 6-OH-apigenin-7-hexoside, thereby reducing oxidative stress. In contrast, for the eucalyptol chemotype, pure red light produced the most significant enhancements in carotenoid synthesis and antioxidant defenses, substantial increases in key compounds such as salvianic, neochlorogenic, rosmarinic, and lithospermic acids, and salvianolic acids E and B, and higher levels of plastochromanol-8. Additionally, both SOD and CAT activities increased, providing greater protection against lipid oxidation. These findings highlight the importance of customizing light treatments not only based on plant species but also according to chemotype to obtain optimal biochemical and physiological outcomes.

## 1. Introduction

*Thymus mastichina* L., commonly known as Spanish marjoram and a member of the *Lamiaceae* family, is endemic to the Iberian Peninsula [1]. This aromatic and medicinal plant is primarily cultivated under rainfed conditions, with a focus on essential oil production. It can be grown as a rainfed crop due to its low water requirements, but nonetheless, may be negatively impacted by climate change-related drought, which can significantly reduce production. Under drought conditions, plants adapt by reducing both stomatal density and leaf size, which helps them minimize water loss and maintain their internal water balance [2]. This reduction in photosynthetic materials leads to a decrease in essential oil yield, as the yield is dependent on both the percentage of essential oil and the overall productivity of the aerial parts of the plant, which are negatively affected when the plant experiences stress [3]. To reduce the negative impacts of drought stress on plants, various strategies have been explored. These include breeding drought-tolerant varieties [4], implementing adaptive practices like cross-adaptation [5], using exogenous substances [6], and modulating light quality through techniques such as priming [7,8]. Research has demonstrated that using red and blue light-emitting diode (LED) lamps significantly enhances the yield of various plant species, outperforming results achieved with broad-spectrum light, which does not effectively provide these wavelength ranges. Specifically, it has been found that red and blue as well as ultraviolet (UV) light can improve several aspects of plant growth, as indicated by higher fresh weight, enhanced photosynthetic activity, greater energy efficiency, and increased levels of essential oils, phenolic compounds, and antioxidants [9]. In line with this, a systematic review conducted by our team previously [10] found that red light spectra applied to plants of *Thymus myrtus*, *Thymus kotschyanus*, and *Thymus carmanicus* increased the essential oil yield and quality.

Key physiological parameters, such as relative water content, antioxidant activity, stomatal behavior, and photosynthetic efficiency, are closely linked to drought tolerance and can be influenced by specific LED light treatments [11]. However, such responses vary significantly between species, highlighting the need for further research to establish optimal light conditions for different crops and production goals [12]. Some aromatic and medicinal plants from the Lamiaceae family, such as rosemary (*Salvia Rosmarinus* L.) and thyme (*Thymus vulgaris* L.), have shown higher growth rates and antioxidant content when grown under red light [9,13]. In contrast, blue light has been found to enhance the growth of basil (*Ocimum basilicum*) by positively affecting photosynthesis, and increase the production of phenolic acids and flavonoids [14]. Nonetheless, it remains uncertain whether monochromatic blue light stimulates the phenylpropanoid pathway through epigenetic mechanisms or alters photosystem II photochemistry via redox signaling in the photosynthetic apparatus [15,16]. In the literature reviewed, no data were found on the effects of LED light on Spanish marjoram production. This study is based on the hypothesis that exposing this species to various spectral ranges of light, specifically white, red, blue, and their combinations may influence its physiological responses and its antioxidant defense system.

## 2. Results

The effects of the six different LED light spectra tested on the two marjoram chemotypes (linalool and eucalyptol) and their interactions are summarized in the analysis of variance results presented in Table 1.

### 2.1. Photosynthetic Pigments (Chlorophyll a, Chlorophyll b, Total Chlorophyll, and Carotenoids)

The analysis of variance results shown in Table 1 indicate that LED light treatments significantly influenced the levels of photosynthetic pigments in both the linalool and eucalyptol chemotypes. Notably, Chl a levels were not affected by light treatment in either ecotype. In contrast, Chl b levels did vary with light treatment though the response depended on the ecotype.

Comparing the effect of LED light between ecotypes, significant differences were found between the spectra (Table 2). Plants with a linalool chemotype grown under white light exhibited the highest Chl T levels. In contrast, the lowest levels of photosynthetic pigments were found in plants exposed to blue (B) light, or white supplemented with B light (C+B). Notably, the synthesis of Chl b seemed to be particularly sensitive to the different light spectra used. When plants were exposed to red (R) light or a combination of red and blue light (R+B), the levels of Chl b were low. Meanwhile, the levels of Chl a were similar to those observed in the control group (C) grown under white light. Additionally, the production of Car was lower under control conditions than with R+B or R light treatments.

In the case of the eucalyptol chemotype, the behavior was different. The Chl T levels were highest with the control treatment and lowest with R+B light. Furthermore, the R light spectrum resulted in the highest levels of carotenoids.

Regarding the Chl a/b ratio, Table 1 indicates no significant differences between the chemotypes. Nonetheless, when examining the specific treatments applied, it was found that the linalool chemotype exhibited the lowest ratio under control conditions, followed by the treatments involving supplementation with red light (C+R) or blue light (C+B). In contrast, the ratio was lowest in the eucalyptol chemotype when exposed to these supplementation treatments (C+R or C+B).

### 2.2. Non-Enzymatic Antioxidants and Lipid Peroxidation

#### 2.2.1. α-Tocopherol and Plastochromanol-8

**Figure 1:**
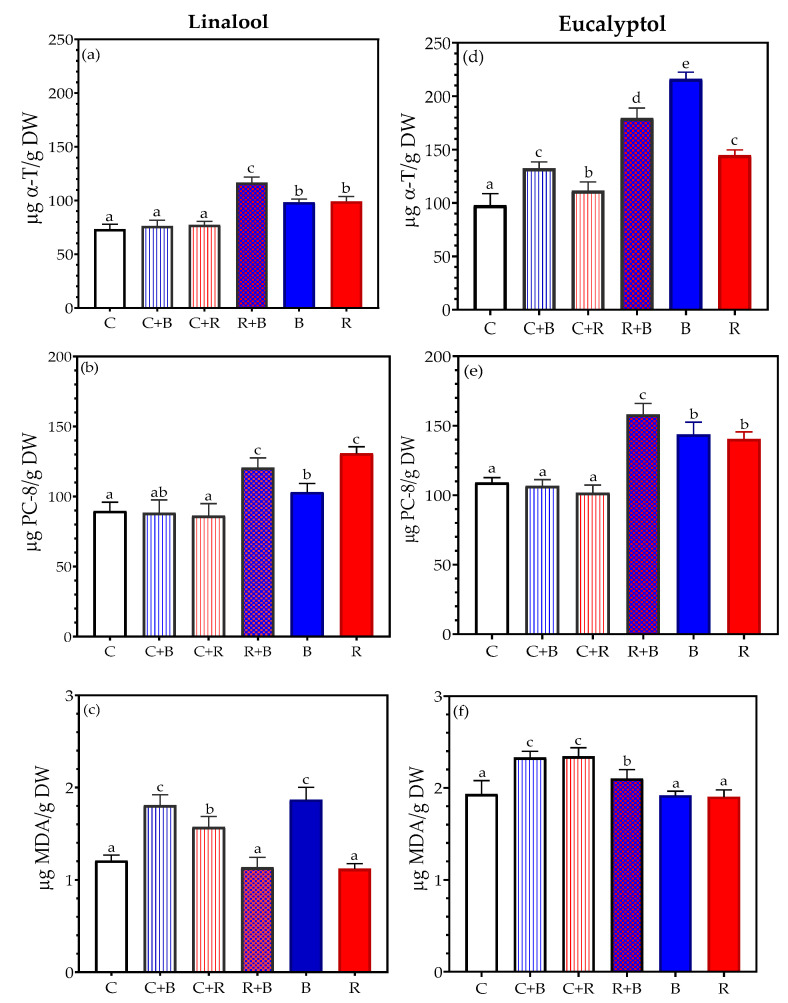
Production of tocochromanols and lipid peroxidation in *Thymus mastichina* under six different light treatments. (**a**,**d**) α-tocopherol, (**b**,**e**) plastochromanol-8, and (**c**,**f**) malondialdehyde. The results are expressed as the mean ± SD. Different letters indicate a significant difference between treatments at *p* < 0.05. Abbreviations: C: control (white light); C+B: white supplemented with blue light; C+R: white supplemented with red light, R+B: combination of red and blue light (70%:30%), B: blue light, and R: red light.

Table 1 indicates significant differences in α-tocopherol (α-T) between chemotypes and also by treatment. Specifically, the data suggest greater synthesis of this tocochromanol in the eucalyptol than the linalool chemotype (Figure 1a,d). Furthermore, there was no consistent pattern in α-T synthesis by treatment in the two chemotypes. Notably, the highest levels of α-T were observed under R+B light in the linalool chemotype, and under the B, followed by R+B light in the case of the eucalyptol chemotype.

Plastochromanol-8 (PC-8) exhibited similar behavior to α-T in both chemotypes (Table 1). Specifically, the highest production of PC-8 was achieved with a combination of R+B light in both types (Figure 1b,e). It is worth noting that the R light treatment also boosted PC-8 levels in the linalool chemotype.

#### 2.2.2. Lipid Peroxidation

In the study of lipid peroxidation, the eucalyptol chemotype demonstrated significantly higher levels of malondialdehyde (MDA) than the linalool chemotype (Table 1). Although there was no consistent pattern of oxidative damage, exposure to C or R light resulted in lower MDA synthesis in both chemotypes (Figure 1). Moreover, Figure 1c,f indicate greater oxidative stress in the linalool chemotype with treatments involving blue light (C+B or B), and in the eucalyptol chemotype with supplementary B or R light (C+B or C+R).

### 2.3. Enzymatic Antioxidant Activity

Table 3 shows that the two chemotypes behaved differently in relation to superoxide dismutase (SOD) activity. Enzyme activity increased in the linalool chemotype with blue light supplementation (C+B), while in the case of the eucalyptol chemotype, it increased significantly with B or R monochromatic light.

In contrast, catalase (CAT) activity results were similar in the two chemotypes (Table 2). This activity increased significantly under R, B, or R+B light, with the highest activity observed under the combined treatment compared to control conditions.

The SOD/CAT ratios were all less than 1, suggesting that plants were able to manage oxidative stress relatively well through enzymatic pathways under all light treatments. In the linalool chemotype, plants exposed to C+R, R+B, B or R light exhibited the lowest ratios, suggesting a superior enzymatic efficiency under these conditions. For the eucalyptol chemotype, a different response was observed. In this case, C+R, B, and R light treatments were associated with a higher SOD/CAT ratio, while the ratio was not notably different with C+B or R+B treatments compared to the control assay.

### 2.4. Polyphenolic Profile and Antioxidant Activity

#### 2.4.1. Polyphenolic Profile

High-performance liquid chromatography (HPLC) diode array detection analysis enabled the identification and quantification of 20 polyphenolic components (Table 4), highlighting both qualitative and quantitative differences between chemotypes and light treatments.

The main differences between the two chemotypes included the absence of luteolin-7-dihexoside, lithospermic acid, and salvianolic acid E in the linalool chemotype, and of rosmarinic-3-O-glucoside in the case of the eucalyptol chemotype. At the quantitative level, the eucalyptol chemotype was found to produce a larger number of polyphenols than the linalool chemotype. In the linalool chemotype, the primary components identified were rosmarinic acid, followed by naringenin and salvianolic acid B. In contrast, the main components in the eucalyptol chemotype were rosmarinic acid, along with lithospermic acid, salvianolic acid E, and the flavonoids 6-OH apigenin-7-hexoside and 6-OH luteolin-7-hexoside.

Regarding the different LED light treatments, the R+B light (at a ratio of 70:30) was found to be the most effective in promoting the accumulation of various hydroxycinnamic acid derivatives and flavones. Specifically, both chemotypes showed increased concentrations of neochlorogenic acid, 4-O-caffeoyl-quinic acid, rosmarinic acid, 6-OH-luteolin-7-hexoside, 6-OH-apigenin-7-hexoside, and 6-OH-luteolin-7-pentoside. In addition, increases were observed in 6-OH-chrysoeriol-7-hexoside, rosmarinic acid 3-O-glucoside, and salvianolic acid B content in the linalool chemotype, and in lithospermic acid and salvianolic acid E in the eucalyptol chemotype.

#### 2.4.2. Antioxidant Activity

**Figure 2:**
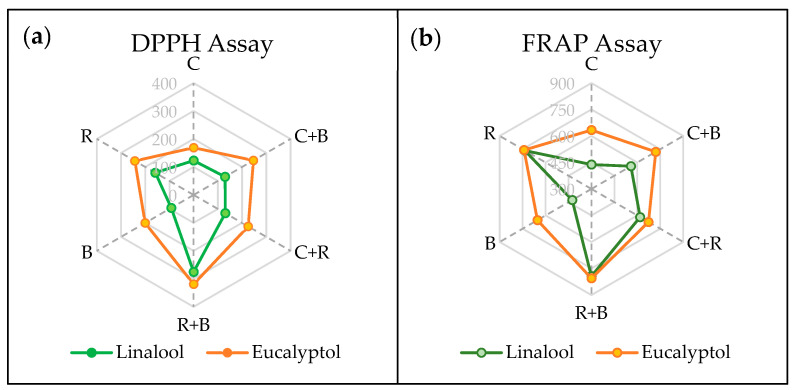
Antioxidant capacity of *Thymus mastichina* under six different light treatments as assessed by (**a**) DPPH^•^ radical scavenging activity (DPPH) and (**b**) ferric-reducing antioxidant power (FRAP) assays. Abbreviations: C: control (white light), C+B: white supplemented with blue light, C+R: white supplemented with red light, R+B: combination of red and blue light (70%:30%), B: blue light, and R: red light.

The antioxidant status of both chemotypes was assessed using two methods: DPPH^•^ radical scavenging activity and ferric-reducing antioxidant power (FRAP), as illustrated in Figure 2. As would be expected, antioxidant activity measured varied depending on the method used.

In terms of antiradical activity, the eucalyptol chemotype generally exhibited greater effectiveness than the linalool chemotype across most treatment conditions; the exception was the R+B treatment, with which both chemotypes demonstrated similarly high radical scavenging activity. In the linalool chemotype, R light was shown to increase the antiradical activity, while exposure to pure B light had a negative effect. In contrast, in the case of plants with the eucalyptol chemotype, exposure to various light spectra had a positive effect, resulting in increased antiradical activity, though the effect was most marked with R+B light.

For the FRAP assay, similar patterns were observed in both chemotypes, with an increase in reducing capacity associated with almost all light treatments, especially R+B and R light. Notably, however, this antioxidant assay indicated a smaller effect with pure B light.

## 3. Discussion

The use of LED lighting in the cultivation of aromatic and medicinal plants has increased due to the potential it offers to customize light spectra. In plants, the perception and absorption of photons promote photomorphogenesis and growth by stimulating photoreceptors and activating associated signaling pathways [17]. This results in changes in gene expression and variations in the biosynthesis of secondary metabolites [18]. These effects are important for increasing commercial production, and specifically, the content of plant-based health-promoting compounds. Therefore, this research focused on the impact of exposure to various light spectra on physiological responses and the antioxidant defense system in two chemotypes of Spanish marjoram.

At a physiological level, the response to the light spectra differed between the genotypes, highlighting the importance of identifying the optimal combination of light spectra based on the species and chemotype being studied [19]. Chlorophyll and carotenoid synthesis was especially sensitive to light quality, with different LED colors influencing the quantity and type of photosynthetic pigments produced.

In both chemotypes, Chl b was more sensitive to light treatments than Chl a, the concentrations measured being lower with almost all the treatments compared to those observed with the control (white light) assay. This type of chlorophyll plays a crucial role in grana stacking, harvesting light at lower intensities, and providing photoprotection [20]. A decrease in this pigment’s content is associated with environmental stress, particularly due to exposure to intense light and high temperatures [21]. However, it is believed that in aromatic plants, light treatments—especially those that combine R and B wavelengths—generally increase Chl b content and overall pigment levels, rather than promoting its degradation [10].

In this context, it is important to consider the Chl a/Chl b ratio, especially when a plant is subjected to changing light conditions, since as chlorophyll content decreases, this ratio tends to increase [22]. Additionally, it can help identify how changes in light conditions affect vegetation. Plants have regulatory mechanisms that can adjust the relative concentrations of Chl a and Chl b in response to stress. These mechanisms may involve alterations in gene expression or enzyme activity, favoring the production or retention of Chl a over Chl b [23]

In the case of the linalool chemotype, the Chl a/Chl b ratio was higher with all treatments compared to the control, though the difference was most pronounced after the R+B, B, and R treatments, which were also associated with greater carotenoid synthesis. In contrast, for the eucalyptol chemotype, the ratio did not differ significantly following the R+B, B, and R light treatments to that observed under control conditions; however, with C+B and C+R treatments, the ratio was significantly lower as was the carotenoid content. It is interesting to note that in both chemotypes, the carotenoid content increased when plants were exposed to R light. According to Nguyen and Sung [24], this spectral range plays a positive role in regulating carotenoid levels through a complex interaction among various transcription factors and genes related to carotenoid biosynthesis. In this context, R light enhances the activity of the enzyme phenylalanine ammonia-lyase, which is crucial for synthesizing phenylpropanoid precursors for various compounds, including some carotenoids [25]. An increase in carotenoid concentration suggests improved photoprotection, helping plants manage oxidative stress due to light exposure.

Plants protect themselves from oxidative stress caused by reactive oxygen species (ROS) through a combination of enzymatic and non-enzymatic antioxidant defense mechanisms. These mechanisms include scavenging ROS, reducing their formation, and repairing oxidative damage to cellular components. [26]. To assess the enzymatic antioxidant system, we considered two enzymes, SOD and CAT. SOD is the primary antioxidant defense enzyme which is present in the cytosol, chloroplasts, peroxisomes, and mitochondria [27], while CAT is located in peroxisomes and mitochondria [28]. The balance between the activities of the antioxidant enzymes is crucial for maintaining cellular health under both normal and stress conditions [27]. While the activities of superoxide dismutase (SOD) and catalase (CAT) can vary based on species and environmental factors, a high SOD to CAT ratio may suggest that a plant is experiencing significant oxidative stress and is relying more on SOD to swiftly neutralize superoxide radicals to prevent cellular damage [27]. We observed this pattern in plants with the linalool chemotype after exposure to white light supplemented with B light (C+B), in which SOD activity was considerably higher than under control conditions, while CAT values did not differ significantly. In contrast, in the eucalyptol chemotype, although higher activity was observed in measurements of both enzymes in plants exposed to R or B light, the balance indicated a smaller increase in CAT with respect to SOD activity.

In this context, in both chemotypes, plants under R+B, B, or R light treatments showed higher levels of CAT than the control group. This scenario usually indicates a change in the plant’s antioxidant defense system, which may lead to increased oxidative stress [29].

In essence, a modification of the SOD and/or CAT activities in aromatic plants can be an indicator of oxidative stress and could potentially impact the plant’s overall health and the quality of its secondary metabolites [30]. Secondary metabolites, such as tocochromanols and polyphenolic components, play a vital role in the antioxidant defense system of plants. Tocochromanols, especially tocopherols, such as α-tocopherol and plastochromanol-8 (located in chloroplasts), are lipid-soluble antioxidants that protect plant membranes from lipid peroxidation and enhance overall stress tolerance [31]. These antioxidants help protect plants from photooxidative stress and lipid peroxidation [32].

Exposure of *Thymus mastichina* to various light spectra seems to increase synthesis of both lipophilic antioxidants. Specifically, in both chemotypes, we noted greater synthesis after exposure to R+B, B, or R light spectra compared to control conditions. These results are in accordance to those published by Trivellini et al. [33], who noticed that exposure to high levels of light, particularly the B and R parts of the spectrum, stimulates the production of these lipophilic antioxidants in chloroplasts. We observed a similar behavior in the accumulation of polyphenols in these shrubs, specifically in the case of hydroxycinnamic acid derivatives and flavones, although this effect was most evident after exposure to R+B light. It is known that applying different light spectra to Thymus species upregulates the expression of key genes such as phenylalanine ammonia-lyase (mentioned above), chalcone synthase, chalcone isomerase, and flavonol synthase, which are central to flavonoid biosynthesis [34].

The light spectrum applied to plants has a significant impact on the levels of various compounds and the specific types of flavonoids and hydroxycinnamic acid derivatives produced. Changes in gene expression indicate these metabolic alterations [35]. For instance, we did not detect compounds such as apigenin-7-glucoside, apigenin-7-O-neohesperidoside, 6-OH-apigenin-7-hexoside, and rosmarinic-3-O-glucoside when B and R+B light spectra were used. On the other hand, R+B light treatment may also significantly increase polyphenolic components, in particular, rosmarinic acid. Its concentration tripled in the linalool chemotype, compared to a twofold increase in the eucalyptol chemotype after exposure to this combination of R+B light.

These variations, specifically those described in the case of the R+B light treatment, improve the plant’s ability to quench ROS and protect cellular components from oxidative damage. In line with this, we observed higher antiradical activity and ferric-reducing power in the methanolic extracts of plants exposed to the combination of these two wavelength ranges (R+B). Higher levels of α-tocopherol and plastochromanol-8, along with salvianolic acid B, rosmarinic acid, and 6-OH-apigenin-7-hexoside, likely account for the greater antioxidant capacities observed [13,36,37,38]. Consequently, less lipid oxidative damage was detected in plants exposed to these light treatments.

Specifically, in the eucalyptol chemotype, the lowest MDA values were measured in plants exposed to monochromatic B or R light spectra. With B light, higher levels of α-T and PC-8 were found, along with higher concentrations of salvianic and lithospermic acids. In the case of the R light exposure, the reduced oxidative damage may be attributed to increased synthesis of PC-8 and rosmarinic acid. Although a higher ratio of SOD to CAT activity was observed in plants exposed to R or B light, both enzymes exhibited increased activity under these treatments compared to the control group. This suggests that a less accumulation of ROS may be associated with a more effective antioxidant defense system in these plants [39].

In the linalool chemotype, the plants exposed to red and blue light (R+B) and R light exhibited the lowest oxidative damage. Notably, the antioxidant role of PC-8 seems to be crucial when compared to α-T, especially in plants treated with R light. PC-8 has previously been identified as a complementary antioxidant to α-T, particularly under stress conditions or when α-T levels are low. It serves as an additional line of defense against lipid peroxyl radicals [40]. Furthermore, the reduced oxidative damage observed is likely also due to the presence of compounds such as salvianolic acid B, rosmarinic acid, and 6-OH-apigenin-7-hexoside, along with the increased activity of CAT. These factors indicate robust plant antioxidant defenses, enabling the plants to better withstand environmental stresses [26].

## 4. Materials and Methods

### 4.1. Plant MaterialIn Vitro Growth and LED Light Treatment

Plant material (*Thymus mastichina* L.) was obtained from the IMIDA germplasm bank, and had been previously selected from wild populations in Lezuza, located in the province of Albacete, and Parandones, in the province of León, Spain. The selection of ecotypes was based on the chemotypes of their essential oils, specifically linalool in one case and eucalyptol in the other. Clones of both ecotypes were multiplied as described by Cáceres-Cevallos et al. [8] with some modifications. First, nodal segments were washed for 18 min in a 20% sodium hypochlorite solution. After that, the explants were rinsed three times with sterilized water. Finally, all explants were placed in Murashige and Skoog medium enriched with vitamins (0.1 mg L^−1^ thiamine HCl, 0.5 mg L^−1^ pyridoxine HCl, 0.5 mg L^−1^ nicotinic acid, and 2.0 mg L^−1^ glycine) and 0.1 mg L^−1^ kinetin, 0.1 mg L^−1^ indole acetic acid and 20.0 g L^−1^ sucrose for 1 month seeking to obtain at least 60 clones from each ecotype. Once the explants had rooted, the seedlings were acclimatized in pots filled with a mixture of peat and perlite (80/20 *w*/*w*) and then placed in the climatic chamber where the light treatments were applied.

The LED light treatments consisted of the exposure of the plants to six different light spectra including white light (photon flux density of approximately 115 μmol/m^2^s, the control condition [C]), white light supplemented with blue light (115 μmol/m^2^s of white and 20 μmol/m^2^s of monochromatic blue [C+B]), white light supplemented with red light (115 μmol/m^2^s of white and 20 μmol/m^2^s of monochromatic red [C+R]), a combination of red and blue light (at a ratio of 70% to 30% providing 115 μmol/m^2^s overall [R+B]), monochromatic blue light (115 μmol/m^2^s [B]), and monochromatic red light (115 μmol/m^2^s [R]). The photosynthetic photon flux density was 115 μmol/m^2^s tested at canopy level and measured using a spectral PAR meter (PG200N, UPRtek, Zhunan Township, Miaoli County, Taiwan). The controlled chamber was kept at 24 °C ± 2 °C, with a relative humidity of 70% to 75% and a 16/8-h light/dark cycle.

After the experiment was finished the plant material was lyophilized and stored at −80 °C until analysis.

### 4.2. Photosynthetic Pigment Extraction and Quantification

Photosynthetic pigments, including chlorophyll a (Chl-a), chlorophyll b (Chl-b), total chlorophyll (Chl-T), and carotenoids (Car), were quantified following the methodology outlined by Szekely-Varga et al. [41] modified. In brief, 50 mg of the lyophilized sample was ground in a mortar with pure methanol and vortexed for two minutes. All samples were then centrifuged at 4 °C at 8400× *g* for three minutes. This process was repeated until the samples turned a whitish color.

All samples were analyzed using a Shimadzu spectrophotometer (UV-2401 PC, Kyoto, Japan) with measurements taken at 665.2 nm for Chl a, 652.4 nm for Chl b, and 470 nm for Car. Photosynthetic pigments were extracted in methanol (pure solvent) and quantified in mg per g of dry weight, using the equations proposed by Lichtenthaler and Buschmann [42].

Chl a (ug/mL) = 16.72 A_665.2_ − 9.16 A_652.4_

Chl b (ug/mL) = 34.09 A_652.4_ − 15.28 A_665.2_

Car (ug/mL) = (1000 A_470_ − 1.63 Chl a − 104.96 Chl b)/221

### 4.3. Non-Enzymatic Antioxidant and Lipid Peroxidation Analysis

#### 4.3.1. α-Tocopherol and Plastochromanol-8 Extraction

Both α-tocopherol (α-T) and plastochromanol-8 (PC-8) were extracted and analyzed using a method modified from Quílez et al. [43]. In brief, 100 mg of plant material was homogenized with 1.5 mL of a cold extraction solution consisting of hexane and ethyl acetate (60/40, *v*/*v*). The mixture was then agitated for 4 h at 15 °C in the dark in an inert atmosphere. To eliminate the water content, anhydrous sodium sulfate was added and the mixture was centrifuged for 10 min at 2061× *g* and 15 °C. The supernatant was then filtered through a 0.45-µm pore size nylon membrane filter (Millipore SAS, Molsheim, France) and evaporated at 35 °C under vacuum using a SpeedVac^®^-SPD121P evaporator system (Thermo Fisher Scientific, Waltham, MA, USA). Finally, the residue was rinsed with 2 mL of pure ethyl acetate and the samples were stored at −80 °C until further analysis by HPLC with fluorescence detector.

The fluorescence analysis was carried out using an HPLC 1200 system (Agilent, Waldbronn, Germany) coupled to a G1311A binary pump, a G1315A photodiode array UV-Vis detector and a G1321A fluorescence detector (excitation at 290 nm and emission at 330 nm). A volume of 15 µL of each sample was injected onto a reverse ZORBAX SB- C18 column (4.6 × 250 mm, 5 µm pore size. Agilent Technologies, Santa Clara, CA, USA) equipped with a guard column ZORBAX SB-C18 (4.6 × 125 mm, 5 µm pore size, Agilent Technologies, Santa Clara, CA, USA), with a flow rate of 1 mL/min at 25 °C.

The mobile phase components for α-T were methanol (phase A) and tert-butyl methyl ether (phase B). Phase B was set to 1% at the start, and subsequently, the gradient applied was as follows: 3 min at 2%; 6 min at 3%; 9 min at 4%; 12 min at 5%; 25 min at 11%; 28 min at 25%; 30 min at 1%; and 35 min at 1%. The mobile phase for PC-8 was based on the method proposed by Gruszka and Kruk [44] with some modifications: setting phase B at 15% initially, and then for 3 min at 45%; 5 min at 55%; 8 min at 65%; 10 min at 15%; and 12 min at 15%. The limits of detection and quantification for both a-T and PC-8 were calculated as described by Cáceres-Cevallos et al. [45].

#### 4.3.2. Lipid Peroxidation Assay

To evaluate the lipid peroxidation, MDA was measured as a byproduct of this process. The analysis was performed according to the methodology described by Cáceres et al. [8] with some modifications. In brief, 100 mg of lyophilized plant was ground in a mortar with 1.0 mL of extraction solution of trichloroacetic acid at 5% (*w*/*v*) and 0.5 mL of butylated hydroxytoluene at 0.8% diluted in hexane (*w*/*v*) to prevent sample oxidation. All samples were centrifuged for 30 min at 4 °C and 20,000× *g*. A 0.4 mL aliquot of the supernatant was mixed with 0.6 mL of thiobarbituric acid at 0.8% (*w*/*v*). Following incubation at 70 °C for 30 min, the samples were placed on ice to stop the reaction, and then centrifuged again at 20,000× *g* and 16 °C for 10 min. Supernatants were placed in vials until HPLC-FLD analysis.

MDA concentrations were measured using the same equipment as that described in Section 4.3.1, with an excitation wavelength of 515 nm and emission wavelength of 553 nm. In brief, a sample volume of 20 µL was injected at a flow rate of 0.8 mL/min and maintained at 35 °C. The mobile phase consisted of acidified water containing 0.05% formic acid (phase A) and methanol (phase B). Phase A was initially set at 95% and then the gradient elution was programmed as follows: 5 min at 75%; 10 min at 5%; 15 min at 5%; 15.1 min at 95%; and finally, 20 min at 95%. The MDA was quantified through linear regression analysis, based on a standard curve obtained using 1,1,3,3-tetraethoxypropane.

### 4.4. Enzymatic Antioxidant Activity Analysis

The enzymes SOD and CAT were extracted using a modified version of the methodology proposed by Zhao et al. [46]. In brief. 0.2 g of lyophilized plant material was homogenized in 2 mL of cold extraction buffer, which consisted of 50 mM potassium phosphate at pH 7.0, 0.5 mM ethylenediaminetetraacetic acid, and 1 mM ascorbic acid. The mixture was then filtered through a 0.44 µm filter and centrifuged at 12,000× *g* for 10 min at 4 °C to remove any debris. Finally, the supernatants were stored at −20 °C until spectrophotometric analysis with the equipment detailed in Section 4.2.

#### 4.4.1. Superoxide Dismutase Activity Assay

SOD activity was measured using a specific assay kit (CS0009, Sigma-Aldrich, Milan, Italy) and measured at a wavelength of 450 nm. The results were reported as units of SOD per mg of dry weight.

#### 4.4.2. Catalase Activity Assay

Similarly, CAT activity was assessed using a specific assay kit (EIACATC, Invitrogen™, Carlsbad, CA, USA) and measured at a wavelength of 560 nm, and the results were expressed as units of CAT per g of dry weight. Additionally, the ratio between SOD and CAT activity was calculated, to estimate the efficacy of these enzymes in counteracting oxidative stress.

### 4.5. Polyphenolic Profile and Antioxidant Activity Analysis

The phenolic compounds were obtained following the method described by Cáceres et al. [45]. In brief, they were extracted from 100 mg of dried sample using 50 mL of methanol in a Soxhlet extractor (B-811) (Buchi, Flawil, Switzerland) for 2 h in a nitrogen atmosphere. The extracts were then dried at 40 °C under vacuum conditions using an evaporator system (Syncore Polyvap R-96; Buchi, Flawil, Switzerland). The residue was re-dissolved in 5 mL of pure methanol. Finally, the extracts were stored in vials at −80 °C until HPLC diode array analysis.

The phenolic profile was analyzed using the HPLC system described in Section 4.3.1. following the method proposed by Jordan et al. [47]. The mobile phase comprised acetonitrile (phase A) and acidified water containing 0.05% formic acid (phase B). Phase B was set to 95% initially and then the gradient employed was: 10 min at 85%; 30 min at 75%; 35 min at 70%; 50 min at 45%; 55 min at 10%; and finally, at 57 min, B was set at 0%, and held for an additional 10 min before returning to the initial conditions. The flow rate was set at 1.0 mL/min, and detection wavelengths at 280 nm, 306 nm, and 330 nm. For quantification, linear regression models were established using standard dilution techniques, and the levels of phenolic compounds were expressed as mg per g of dry weight.

### 4.6. Antioxidant Capacity Testing

Antioxidant activity was assessed using both the FRAP and DPPH assays. The FRAP assay was conducted following a modified version of the method proposed by Szabó et al. [48]. Briefly, the FRAP reagent was prepared by mixing 300 mM acetate buffer (pH 3.6), 10 mM 2,4,6-tripyridyl-s-triazine dissolved in 40 mM HCl, and 20 mM FeCl_3_ at a ratio of 10:1:1 (*v*/*v*/*v*). An aliquot of 40 µL was combined with 1.2 mL of the FRAP reagent and incubated for 30 min in the dark at 37 °C. All samples were then measured spectrophotometrically at a wavelength of 593 nm. The results were expressed as µmol of Fe^2+^ per g of dry plant material.

The DPPH analysis was conducted using the method outlined by Farhat et al. [49]. In brief, 500 µL of the extracts were combined with 1 mL of a 0.1 mM DPPH methanolic solution. The samples were then measured with a spectrophotometer at a wavelength of 517 nm after being incubated for 20 min at room temperature in the dark. Absorbance readings were taken against a blank consisting of 500 µL of sample and 1 mL of methanol. The results were expressed as µM of Trolox equivalents per g of dry plant.

### 4.7. Statistical Analysis

Data are presented as mean ± standard deviation based on the number of replicates. The homoscedasticity assumption was first tested using Levene’s test. Then, one- and two-way analyses of variance were conducted, followed by Tukey’s post hoc test. The relationships among variables were assessed by calculating Pearson’s correlation coefficients. The statistical analysis was carried out using STATGRAPHICS Centurion XVI.I and GraphPad Prism 8 software.

## 5. Conclusions

The study examined how different LED light spectra influence the synthesis of photosynthetic pigments and the modulation of the antioxidant defense system in two ecotypes of Spanish marjoram with different chemotypes. The results revealed that the responses varied depending on the wavelengths of light used and the chemotype studied.

For the linalool chemotype, the treatment that resulted in the least oxidative damage, compared to the control group, involved a combination of red and blue light at a 70:30 ratio (R+B). Under these conditions, the concentration of carotenoids increased, indicating enhanced photoprotection. Additionally, an increase was observed in antioxidant capacity, which correlated with a greater accumulation of PC-8 and phenolic compounds, including salvianolic acid B, rosmarinic acid, and 6-OH-apigenin-7-hexoside. There was also a significant rise in catalase activity, highlighting the strengthening of the antioxidant defense system in this chemotype.

In the eucalyptol chemotype, while the highest antioxidant capacities were achieved using the R+B light treatment, the lowest levels of oxidative damage, similar to those in the control group, were recorded in plants exposed to either blue or red light spectra. Notably, the R treatment obtained better results in terms of modulating photosynthetic pigment synthesis and enhancing the antioxidant defense system. Compared to results under white light, this treatment was associated with higher levels of PC-8, as well as higher concentrations of salvianic acid, neochlorogenic acid, rosmarinic acid, lithospermic acid, and both salvianolic acids E and B. A higher SOD to CAT ratio was also observed, with both enzymes showing increased activity. This suggests that exposing this chemotype to red light enhances the plants’ antioxidant system, improving their antioxidant capacity and preventing lipid oxidative damage.

Future studies could explore the molecular mechanisms behind this effect and examine its impact under different environmental conditions. Additionally, these results could inform agricultural and horticultural practices, supporting the use of light treatments to enhance crop quality and stress resilience.

## Figures and Tables

**Table 1:** Analysis of variance of chlorophyll a and b content, total chlorophyll content and the a/b ratio (Chl a, Chl b, Chl T, and Chl a/b respectively), as well as carotenoid (Car), α-tocopherol (α-T), plastochromanol-8 (PC-8), and malondialdehyde (MDA) content in two ecotypes of *Thymus mastichina* under six different light treatments, and the chemotype-treatment interaction.

			Mean Squares						
Source of Variation	df	Chl a	Chl b	Chl T	Car	Chl a/b	α-T	PC-8	MDA
Chemotype under light treatment (linalool or eucalyptol)	1	32.13 ***	3.70 *	81.18 ***	4.20 ***	0.001	28968 ***	3558 *	4.2 ***
Light treatment type (six different spectra)	5	3.28	1.41 *	11.47 *	0.45 *	0.184 ***	5104 **	3452 ***	0.34
Chemotype x light treatment (A×B)	5	3.03	2.32 ***	8.74	0.32	0.111 *	1622	311.9	0.22

*, **, and *** indicate significance at *p* < 0.05, *p* < 0.01, and *p* < 0.001, respectively.

**Table 2:** Effects of six different light spectra on the content of plant pigments, namely, chlorophyll-a (Chl a), chlorophyll-b (Chl b), total chlorophyll (Chl T), and carotenoids (Car).

		Treatment					
Chemotype	Pigment	C	C+B	C+R	R+B	B	R
*Thymus* *mastichina* (linalool)	Chl a	8.3 ± 0.4 ^c^	5.8 ± 0.2 ^a^	7.5 ± 0.4 ^b^	8.1 ± 0.3 ^c^	6.0 ± 0.4 ^a^	8.1 ± 0.5 ^bc^
	Chl b	5.4 ± 0.4 ^c^	2.6 ± 0.2 ^a^	3.7 ± 0.2 ^b^	3.6 ± 0.3 ^b^	2.7 ± 0.3 ^a^	3.4 ± 0.4 ^b^
	Car	1.5 ± 0.1 ^b^	1.3 ± 0.1 ^a^	1.6 ± 0.1 ^b^	1.9 ± 0.1 ^c^	1.4 ± 0.1 ^a^	2.0 ± 0.1 ^c^
	Chl T	13.4 ± 0.6 ^c^	8.2 ± 0.4 ^a^	11.1 ± 0.6 ^b^	11.5 ± 0.2 ^b^	8.5 ± 0.8 ^a^	11.6 ± 0.8 ^b^
	Chl a/b	1.7 ± 0.04 ^a^	2.2 ± 0.1 ^b^	2.0 ± 0.02 ^b^	2.3 ± 0.2 ^c^	2.2 ± 0.1 ^c^	2.3 ± 0.1 ^c^
*Thymus* *mastichina* (eucalyptol)	Chl a	9.9 ± 0.2 ^c^	9.0 ± 0.3 ^b^	8.1 ± 0.2 ^a^	8.2 ± 0.2 ^a^	9.2 ± 0.1 ^b^	9.7 ± 0.3 ^c^
	Chl b	4.1 ± 0.1 ^bc^	4.6 ± 0.1 ^e^	4.3 ± 0.2 ^cd^	3.7 ± 0.1 ^a^	4.0 ± 0.1 ^b^	4.4 ± 0.2 ^de^
	Car	2.3 ± 0.1 ^c^	1.9 ± 0.1 ^b^	1.7 ± 0.1 ^a^	2.0 ± 0.1 ^b^	2.3 ± 0.2 ^c^	2.7 ± 0.1 ^d^
	Chl T	14.4 ± 0.3 ^d^	13.4 ± 0.4 ^bc^	12.8 ± 0.7 ^ab^	12.1 ± 0.5 ^a^	13.2 ± 0.1 ^b^	14.1 ± 0.5 ^d^
	Chl a/b	2.2 ± 0.1 ^cd^	1.9 ± 0.01 ^ab^	1.8 ± 0.2 ^a^	2.0 ± 0.2 ^bc^	2.3 ± 0.1 ^cd^	2.2 ± 0.03 ^d^

For each component, different letters indicate a significant difference between light treatments at *p* < 0.05. Abbreviations: C: control (white light), C+B: white supplemented with blue light, C+R: white supplemented with red light, R+B: combination of red and blue light (70%:30%), B: blue light, and R: red light.

**Table 3:** Effects of six different light treatments on antioxidant activities, as indicated by superoxide dismutase (SOD) and catalase (CAT) per g of fresh weight and the SOD/CAT ratio.

		Treatments					
Chemotypes	Enzymes	C	C+B	C+R	R+B	B	R
*Thymus* *mastichina* (linalool)	SOD	161.3 ± 19.3 ^a^	247.3 ± 17.2 ^b^	164.8 ± 28.3 ^a^	169.2 ± 26.7 ^a^	152.1 ± 20.6 ^a^	162.2 ± 31.0 ^a^
	CAT	351.6 ± 26.9 ^a^	372.4 ± 9.8 ^ab^	402.5 ± 17.4 ^b^	530.1 ± 29.2 ^d^	452.3 ± 26.6 ^c^	496.6 ± 20.4 ^d^
	SOD/CAT	0.5 ± 0.03 ^b^	0.7 ± 0.04 ^c^	0.4 ± 0.07 ^ab^	0.3 ± 0.04 ^a^	0.3 ± 0.05 ^a^	0.3 ± 0.07 ^a^
*Thymus* *mastichina* (eucalyptol)	SOD	147.7 ± 18.3 ^a^	134.6 ± 19.2 ^a^	220.8 ± 22.0 ^b^	145.3 ± 24.7 ^a^	303.1 ± 23.4 ^c^	325.3 ± 16.6 ^c^
	CAT	452.6 ± 26.8 ^a^	458.9 ± 27.3 ^a^	540.2 ± 20.7 ^b^	659.9 ± 20.0 ^c^	645.5 ± 20.2 ^c^	637.3 ± 26.4 ^c^
	SOD/CAT	0.3 ± 0.03 ^b^	0.3 ± 0.06 ^ab^	0.4 ± 0.04 ^c^	0.2 ± 0.03 ^a^	0.5 ± 0.03 ^cd^	0.5 ± 0.04 ^d^

For each component, different letters indicate a significant difference between light treatments at *p* < 0.05. Abbreviations: C: control (white light), C+B: white supplemented with blue light, C+R: white supplemented with red light, R+B: combination of red and blue light (70%:30%), B: blue light, and R: red light.

**Table 4:** Quantitative phenolic profile of *Thymus mastichina* and its response to six different light treatments.

		Treatments					
Phenolic Compound	Chemotype	C	C+B	C+R	R+B	B	R
Salvianic acid	Linalool	0.06 ± 0.005 ^b^	0.07 ± 0.008 ^c^	0.06 ± 0.008 ^bc^	0.06 ± 0.006 ^b^	0.06 ± 0.006 ^bc^	0.04 ± 0.004 ^a^
	Eucalyptol	0.07 ± 0.002 ^a^	0.08 ± 0.005 ^a^	0.07 ± 0.005 ^a^	0.09 ± 0.008 ^b^	0.14 ± 0.009 ^c^	0.10 ± 0.004 ^b^
Neochlorogenic acid	Linalool	0.03 ± 0.01 ^a^	0.04 ± 0.01 ^a^	0.04 ± 0.01 ^a^	0.09 ± 0.01 ^c^	0.03 ± 0.01 ^a^	0.07 ± 0.01 ^b^
	Eucalyptol	0.03 ± 0.01 ^a^	0.03 ± 0.01 ^a^	0.03 ± 0.01 ^ab^	0.06 ± 0.01 ^c^	0.05 ± 0.01 ^b^	0.05 ± 0.01 ^b^
4-O-caffeoyl-quinic acid	Linalool	0.3 ± 0.04 ^b^	0.3 ± 0.04 ^b^	0.3 ± 0.03 ^b^	0.4 ± 0.03 ^c^	0.2 ± 0.01 ^a^	0.3 ± 0.01 ^b^
	Eucalyptol	0.2 ± 0.04 ^a^	0.2 ± 0.03 ^a^	0.2 ± 0.02 ^a^	0.4 ± 0.04 ^b^	0.3 ± 0.03 ^a^	0.2 ± 0.01 ^a^
Caffeic acid	Linalool	0.3 ± 0.04 ^c^	0.4 ± 0.02 ^c^	0.3 ± 0.01 ^b^	0.2 ± 0.01 ^a^	0.2 ± 0.03 ^b^	0.2 ± 0.02 ^b^
	Eucalyptol	0.2 ± 0.02 ^ab^	0.2 ± 0.02 ^bc^	0.2 ± 0.01 ^ab^	0.2 ± 0.01 ^c^	0.2 ± 0.01 ^b^	0.2 ± 0.01 ^a^
4-O-feruloylquinic acid	Linalool	0.3 ± 0.02 ^b^	0.4 ± 0.03 ^c^	0.3 ± 0.04 ^ab^	0.3 ± 0.03 ^bc^	0.3 ± 0.03 ^ab^	0.3 ± 0.02 ^a^
	Eucalyptol	0.2 ± 0.03 ^b^	0.2 ± 0.02 ^a^	0.2 ± 0.01 ^a^	0.3 ± 0.02 ^b^	0.3 ± 0.02 ^b^	0.2 ± 0.02 ^b^
6-OH-luteolin-7-hexoside	Linalool	0.3 ± 0.04 ^b^	0.3 ± 0.04 ^b^	0.4 ± 0.03 ^b^	0.5 ± 0.03 ^c^	0.2 ± 0.02 ^a^	0.3 ± 0.03 ^a^
	Eucalyptol	1.2 ± 0.09 ^b^	2.1 ± 0.13 ^d^	2.0 ± 0.10 ^d^	1.7 ± 0.15 ^c^	0.9 ± 0.06 ^a^	1.1 ± 0.13 ^ab^
Luteolin-7-dihexoside	Linalool	n.d.	n.d.	n.d.	n.d.	n.d.	n.d.
	Eucalyptol	0.1 ± 0.02 ^ab^	0.2 ± 0.02 ^d^	0.2 ± 0.01 ^cd^	0.2 ± 0.02 ^bc^	0.1 ± 0.01 ^a^	0.1 ± 0.01 ^a^
6-OH-chrysoeriol-7-hexoside	Linalool	0.04 ± 0.01 ^a^	0.06 ± 0.01 ^bc^	0.06 ± 0.01 ^c^	0.06 ± 0.01 ^bc^	0.06 ± 0.01 ^c^	0.05 ± 0.01 ^ab^
	Eucalyptol	0.1 ± 0.02 ^a^	0.1 ± 0.01 ^c^	0.1 ± 0.00 ^c^	0.1 ± 0.01 ^c^	0.1 ± 0.01 ^b^	0.1 ± 0.01 ^a^
6-OH-apigenin-7-hexoside	Linalool	0.7 ± 0.02 ^a^	0.7 ± 0.09 ^a^	0.7 ± 0.11 ^a^	1.2 ± 0.10 ^b^	n.d.	0.8 ± 0.08 ^a^
	Eucalyptol	2.0 ± 0.09 ^ab^	3.2 ± 0.29 ^d^	3.3 ± 0.20 d	2.6 ± 0.11 c	1.7 ± 0.10 a	2.2 ± 0.14 ^b^
Rosmarinic-3-O-glucoside	Linalool	0.2 ± 0.03 ^a^	0.3 ± 0.02 ^b^	0.2 ± 0.04 ^a^	0.4 ± 0.02 ^c^	n.d.	0.3 ± 0.02 ^b^
	Eucalyptol	n.d.	n.d.	n.d.	n.d.	n.d.	n.d.
6-OH-luteolin-7-pentoside	Linalool	0.3 ± 0.02 ^a^	0.4 ± 0.09 ^b^	0.4 ± 0.03 ^b^	1.0 ± 0.09 ^d^	0.5 ± 0.04 ^b^	0.6 ± 0.05 ^c^
	Eucalyptol	0.4 ± 0.00 ^b^	0.7 ± 0.04 ^d^	0.7 ± 0.03 ^d^	0.8 ± 0.03 ^e^	0.5 ± 0.03 ^c^	0.4 ± 0.03 ^a^
Apigenin-7-O-neohesperidoside	Linalool	0.1 ± 0.01	0.1 ± 0.03	0.1 ± 0.03	n.d.	n.d.	0.1 ± 0.01
	Eucalyptol	0.1 ± 0.01 ^a^	0.1 ± 0.01 ^a^	0.2 ± 0.04 ^b^	0.1 ± 0.01 ^a^	0.1 ± 0.02 ^a^	0.1 ± 0.02 ^a^
Apigenin-7-glucoside	Linalool	0.4 ± 0.03 ^b^	n.d.	0.7 ± 0.10 ^d^	n.d.	0.5 ± 0.03 ^c^	0.3 ± 0.04 ^a^
	Eucalyptol	0.3 ± 0.03 ^a^	0.5 ± 0.05 ^b^	0.8 ± 0.06 ^c^	0.6 ± 0.08 ^b^	n.d.	0.3 ± 0.09 ^a^
Rosmarinic acid	Linalool	10.6 ± 1.8 ^a^	13.9 ± 1.7 ^b^	12.4 ± 1.9 ^ab^	33.5 ± 2.3 ^d^	12.3 ± 2.1 ^ab^	22.3 ± 2.0 ^c^
	Eucalyptol	11.7 ± 1.1 ^a^	12.9 ± 1.9 ^a^	12.8 ± 1.5 ^a^	20.9 ± 2.3 ^c^	12.9 ± 1.6 ^a^	15.8 ± 1.0 ^b^
Lithospermic acid	Linalool	n.d.	n.d.	n.d.	n.d.	n.d.	n.d.
	Eucalyptol	2.4 ± 0.17 ^a^	2.8 ± 0.20 ^b^	2.8 ± 0.12 ^b^	3.1 ± 0.14 ^c^	3.6 ± 0.10 ^d^	2.9 ± 0.10 ^bc^
Salvianolic acid E	Linalool	n.d.	n.d.	n.d.	n.d.	n.d.	n.d.
	Eucalyptol	2.1 ± 0.18 ^a^	2.4 ± 0.16 ^ab^	2.7 ± 0.11 ^c^	3.3 ± 0.08 ^d^	2.2 ± 0.13 ^ab^	2.4 ± 0.17 ^b^
Salvianolic acid B	Linalool	0.7 ± 0.02 ^b^	0.7 ± 0.05 ^b^	0.7 ± 0.02 ^b^	1.1 ± 0.04 ^d^	0.6 ± 0.02 ^a^	0.8 ± 0.02 ^c^
	Eucalyptol	0.8 ± 0.06 ^a^	1.0 ± 0.05 ^b^	1.1 ± 0.07 ^b^	1.0 ± 0.09 ^b^	1.0 ± 0.06 ^b^	1.0 ± 0.06 ^b^
Eriodyctiol	Linalool	0.2 ± 0.01 ^d^	0.2 ± 0.03 ^cd^	0.1 ± 0.01 ^b^	0.2 ± 0.01 ^bc^	0.1 ± 0.02 ^bc^	0.1 ± 0.02 ^a^
	Eucalyptol	0.2 ± 0.03 ^b^	0.4 ± 0.03 ^d^	0.3 ± 0.02 ^c^	0.2 ± 0.02 ^b^	0.2 ± 0.03 ^a^	0.2 ± 0.02 ^b^
Naringenin	Linalool	0.8 ± 0.04 ^e^	0.7 ± 0.03 ^d^	0.6 ± 0.04 ^c^	0.3 ± 0.04 ^a^	0.5 ± 0.03 ^b^	0.4 ± 0.02 ^b^
	Eucalyptol	0.3 ± 0.02 ^d^	0.3 ± 0.01 ^bc^	0.2 ± 0.02 ^a^	0.2 ± 0.04 ^a^	0.3 ± 0.03 ^cd^	0.3 ± 0.03 ^b^
Pinocembrin	Linalool	0.3 ± 0.02 ^b^	0.3 ± 0.02 ^b^	0.2 ± 0.01 ^ab^	0.2 ± 0.02 ^ab^	0.2 ± 0.02 ^ab^	0.2 ± 0.01 ^a^
	Eucalyptol	0.2 ± 0.02 ^b^	0.2 ± 0.01 ^b^	0.2 ± 0.02 ^a^	0.2 ± 0.01 ^ab^	0.2 ± 0.02 ^c^	0.2 ± 0.02 ^bc^

For each component, different letters indicate a significant difference between light treatments at *p* < 0.05. Abbreviations: C: control (white light), C+B: white supplemented with blue light, C+R: white supplemented with red light, R+B: combination of red and blue light (70%:30%), B: blue light, R: red light, and n.d.: not detected.

## Data Availability

The original contributions presented in this study are included in the article. Further inquiries can be directed to the corresponding author.

## Abbreviations

The following abbreviations are used in this manuscript:

SOD	Superoxide dismutase
CAT	Catalase
LED	Light-emitting diode
Chl a	Chlorophyll a
Chl b	Chlorophyll b
Chl T	Total chlorophylls
Car	Carotenoids
α-T	α-Tocopherol
PC-8	Plastochromanol-8
MDA	Malondialdehyde
FRAP	Ferric Reducing Antioxidant Power
DPPH	2,2′-diphenyl-1-picrylhydrazyl
